# To bnAb or Not to bnAb: Defining Broadly Neutralising Antibodies Against HIV-1

**DOI:** 10.3389/fimmu.2021.708227

**Published:** 2021-10-19

**Authors:** Sarah A. Griffith, Laura E. McCoy

**Affiliations:** Division of Infection and Immunity, Institute of Immunity and Transplantation, University College London, London, United Kingdom

**Keywords:** HIV-1, broadly neutralising antibody, somatic hypermutation, complementary determining region, epitope

## Abstract

Since their discovery, antibodies capable of broad neutralisation have been at the forefront of HIV-1 research and are of particular interest due to *in vivo* passive transfer studies demonstrating their potential to provide protection. Currently an exact definition of what is required for a monoclonal antibody to be classed as a broadly neutralising antibody (bnAb) has not yet been established. This has led to hundreds of antibodies with varying neutralisation breadth being studied and has given insight into antibody maturation pathways and epitopes targeted. However, even with this knowledge, immunisation studies and vaccination trials to date have had limited success in eliciting antibodies with neutralisation breadth. For this reason there is a growing need to identify factors specifically associated with bnAb development, yet to do this a set of criteria is necessary to distinguish bnAbs from non-bnAbs. This review aims to define what it means to be a HIV-1 bnAb by comparing neutralisation breadth, genetic features and epitopes of bnAbs, and in the process highlights the challenges of comparing the array of antibodies that have been isolated over the years.

## Introduction

The only antigen exposed on the surface of human immunodeficiency virus (HIV)-1 is the envelope glycoprotein (Env), a trimer comprised of cleaved gp120-gp41 heterodimers, and is located on the surface of HIV to mediate entry into cells (1, 2). Neutralising antibodies (nAbs) directed towards Env can block viral entry and prevent infection by interfering with engagement of host cell receptors (CD4) or co-receptors (CCR5 or CXCR4), by stabilising pre-fusion Env to prevent membrane fusion or by increasing Env decay (3–9). Initial strain-specific nAbs, produced by the majority of HIV-1 infected individuals, can constrict infection and thus exert selection pressure on the virus (10, 11). However, viruses that have already entered cells integrate their genome into the host genome using a viral-encoded reverse transcriptase that is highly error-prone, with a mutation rate of 3.4 x 10^-5^ per base during a single round of replication (12). Random mutations introduced into the envelope gene (*env*) during replication may remove or hinder access to the epitopes targeted by nAbs, leading to neutralisation resistant variants that are able to persist and continue to infect new cells (13). High mutation rates combined with a short replication cycle and a tendency for recombination consequently results in increasing quasispecies and viral diversity within an individual over the course of HIV-1 infection (14). After a few years some individuals infected with HIV-1 (10-30% adults) can develop nAbs capable of cross-neutralisation, and an even smaller subset (1-10%) termed elite neutralisers are able to produce broadly neutralising antibodies (bnAbs) (15). Infants on the other hand have a distinct immune response and often develop broadly neutralising plasma after as early as one year post-infection (16). This has been attributed to multivariant infection as opposed to diversity driven by viral escape due to the finding that circulating viruses in elite neutralisers are still sensitive to autologous nAbs in the plasma (17). Individual bnAbs isolated from HIV-infected individuals are currently of particular interest because passively transferred bnAbs can provide *in vivo* protection in animal models (18–22) and suppression of viral rebound in humans (23). This suggests a key role for bnAbs in the control of new and pre-existing HIV-1 infections. Moreover, these findings give support to the idea that a vaccine capable of eliciting bnAbs could provide the immune system with the head start required to prevent HIV-1 infection.

The first bnAbs were discovered in the early 1990’s, and since then over 300 antibodies described as bnAbs and their lineage members have been isolated (24). These have been extensively studied to investigate their development, structural and genetic features, as well as the epitopes that they target. Poly-reactivity and auto-reactivity are common features among some bnAbs and have shown association with the ability to neutralise HIV (25, 26). Extreme somatic hypermutation (SHM) is a trait that has also been observed among the majority of bnAbs isolated from adults (27), and in some cases antibodies feature large insertions and/or deletions too, suggesting that complex affinity maturation pathways drive their development. Another characteristic trait is the length of time required for bnAbs to develop in infected adults, usually taking up to 2-3 years after original HIV-1 exposure, but in some cases can take up to 5 years to develop (28–33). The acquisition of high levels of SHM and slow development of these antibodies can be explained by the evolutionary arms race between the humoral immune response and HIV. Initial nAbs exert selection pressure on the virus that leads to viral variants capable of escaping neutralisation, this in turn selects for affinity-matured antibodies that again exert pressure on the virus and thus results in an ongoing cycle regarded as an evolutionary arms race (34). Ultimately, breadth of neutralisation is achieved by nAbs targeting conserved sites on the functional trimeric Env such as the CD4 binding site, trimer apex, high-mannose patch, gp120-gp41 interface (including the fusion peptide), membrane proximal region (MPER) and the more recently identified epitope referred to as the ‘silent face’ (35). The main bnAb epitopes, their location on the Env trimer and the mechanism of neutralisation by bnAbs have been brilliantly illustrated in existing reviews (36, 37). As mentioned in these reviews, the Env trimer is a glycoprotein covered by a high density of glycans that are added by the host post-translation according to the N-linked glycosylation sites encoded in the viral sequence (N-X-S/T). These N-linked glycans on Env consist of ~50% of the gp120 mass (38) and are generally less immunogenic than the protein itself. Although the presence of glycans lead to sites on Env being shielded from nAb access (39), the majority of bnAbs are in fact able to accommodate or even incorporate glycans in their epitopes (36). Furthermore, many bnAbs utilise infrequent genetic features such as long heavy chain complementary determining regions (CDRs) (40, 41), that are favourable to access recessed epitopes on Env. Overall, the genetic and structural features of bnAbs are unusual for antibodies, and have been suggested to be a result of perturbations in the regulation of immune tolerance mechanisms during chronic infection and inflammation (42–44).

As described above, individuals with sera that demonstrate very broad neutralising activity are termed elite neutralisers. This can be defined by the ability to neutralise a minimum of one Env pseudo-typed virus (PV) with an IC_50_ titre of 300, across four clades (45). The authors who defined this criterion also proposed a system by which a neutralisation score can be calculated to rank and characterise sera (average log-transformed titres for a PV panel), with scores ≥ 2.5 indicating elite neutralisers capable of producing bnAbs (45). Furthermore, this scoring system has proven successful with follow up studies confirming that bnAbs can be isolated from donors identified as elite neutralisers (46, 47). Currently HIV bnAbs are described as antibodies which; are highly effective against most circulating strains, neutralise a wide range of genetically diverse HIV-1 subtypes, potently neutralise a substantial percentage of primary isolates or exhibit some capacity to reach across clades and harder to neutralise tier 2 and 3 viruses (34, 36, 48, 49). Whilst these descriptions highlight the broad reactivity and potency of bnAbs, they are also vague and do not define a set of criteria to distinguish between an antibody that is cross-neutralising to a degree or a true bnAb. This raises the question: what specific requirements need to be met for an antibody to be classed as a bnAb? This could be answered by defining a certain percentage of strains that need to be neutralised by a bnAb, however the challenge lies in determining where the threshold should be. Over the years the neutralising activities of many HIV antibodies have been tested against different PVs in different studies. A major caveat is that these studies vary not only in the number of viruses tested but also in the types of viruses included in panels, making it very hard to compare neutralisation breadth. However the development of a tiering system which characterises the sensitivity of a virus to antibody neutralisation and establishment of reference viruses has greatly improved the systematic screening of nAb responses (50). Another factor that could be considered to define what is or isn’t a bnAb is neutralisation potency. This characteristic reflects how effective an antibody is at inhibiting viral infection and has previously been used to show differences between the first and second generations of bnAbs (37). An alternative criteria to define a bnAb could plausibly be found among the unusual structural or genetic features of HIV bnAbs. However, these features appear to vary depending on the epitope targeted, again making a precise definition difficult. Until now, that the field has not converged on a conclusive set of criteria for what constitutes a bnAb has arguably been beneficial. Hundreds of antibodies have been isolated that have given insight into the exact epitopes targeted by different bnAb classes and the developmental pathways of bnAbs have been investigated by studying lineage members. Many of these antibodies may have been missed or excluded if there had been a strict bnAb cut-off point. Nevertheless with most attempts to elicit a bnAb response in immunisation studies and vaccine trials being unsuccessful (51, 52), there is an increasing need to define what constitutes a bnAb in order to investigate the host immune responses associated with bnAb development (53–56). Studies to date have only made distinctions between bnAb and non-nAb responses at the level of serum neutralisation (28, 54, 57–59), a caveat being that this is a polyclonal response. It would therefore be valuable to have a clear division at the monoclonal antibody level to categorise bnAbs from non-bnAbs, which can only be achieved by defining what it means to be a bnAb.

This review will focus on a subset of monoclonal antibodies isolated from HIV-1 infected adults that have been previously tested for neutralisation and subsequently referred to as bnAbs. The Los Alamos National Lab (LANL) HIV database (24) was used to select a range of these antibodies from different donors and lineages. In addition, a literature search for HIV-1 broadly neutralising antibodies, restricted to papers published from 2018-2021, was also conducted to include more recently isolated antibodies not yet listed on the LANL HIV database.

## Can We Define HIV bnAbs by Comparing Their Neutralisation Profiles?

The first generation of antibodies against HIV-1 termed as bnAbs were isolated prior to 2009 using phage display and hybridoma technology, since then advances in methods to generate and assess monoclonal antibodies (mAbs) led to a second generation of more potent bnAbs being isolated *via* single B cell cloning following either single B cell culture or antigen-specific sorting [reviewed in (36)]. More recently, a novel technique utilising a matched genomic and proteomic approach has been used to deconvolute polyclonal plasma and successfully isolate bnAb lineages (30). Following isolation, antibodies are screened against PVs in assays to determine their neutralisation capacity (60, 61). However, due to the diversity of HIV-1 there is a vast array of *env* variants that can be pseudotyped for use in these assays. Therefore it is no surprise that the author-defined neutralisation breadth highlighted in **Supplementary Table 1** has been assessed using different virus panels. This makes the comparison of antibody breadth problematic, and to add complexity to this matter, the number of viruses within each panel vary as do the clades and tiers of viruses included.

## The Impact of Viral Tiers and Clades on Neutralisation Profiles of Antibodies

**Figure 1:**
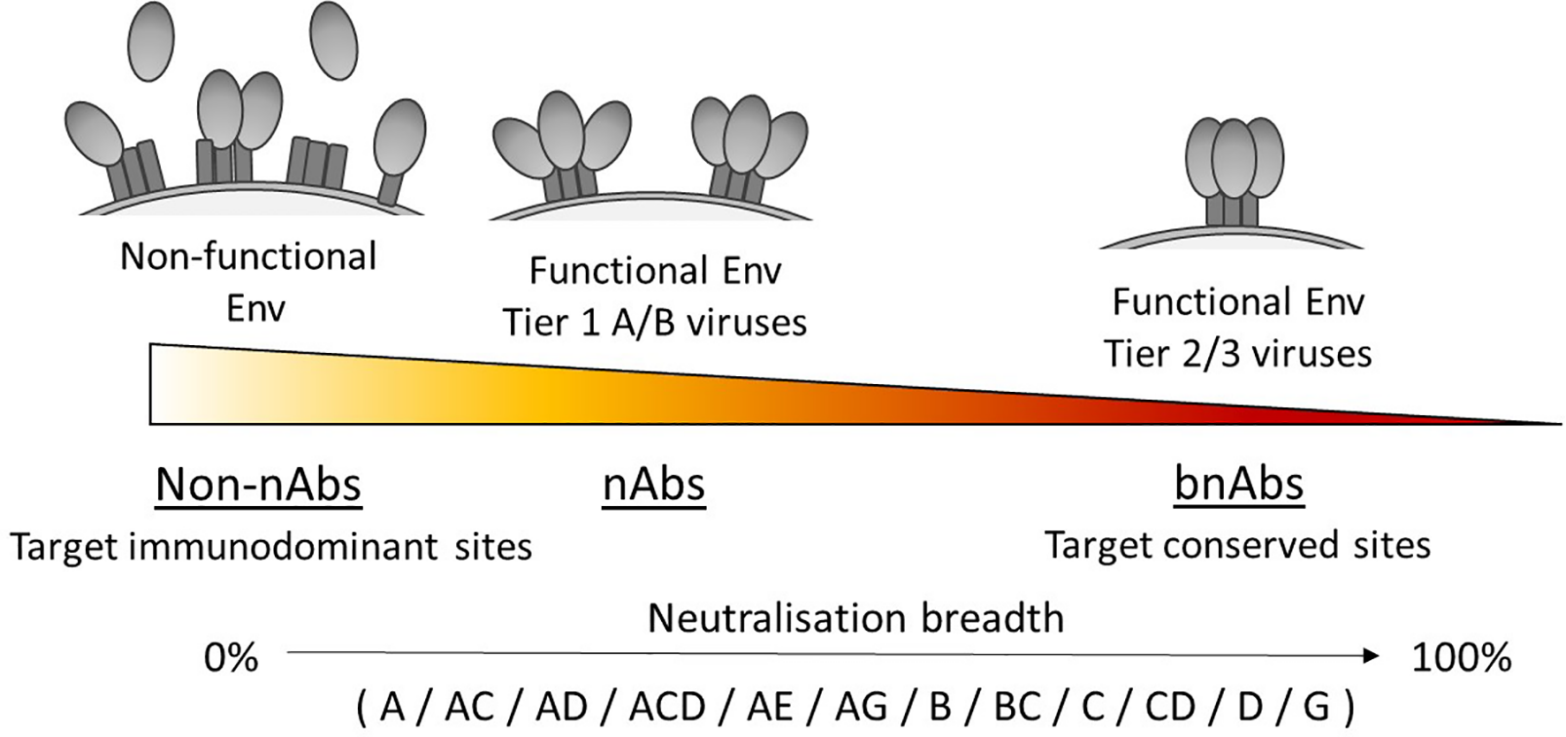
Neutralisation breadth corresponds with the ability to target functional Env trimers in a closed conformation. Tier 1A, tier 1B and tier 2/3 viruses have a predominantly open, intermediate and closed Env trimer conformation respectively and relates to their susceptibility to neutralisation, with tier 2/3 viruses being harder to neutralise. Antibodies that can neutralise tier 2/3 viruses from multiple clades (listed here in alphabetical order) have increased breadth by targeting conserved sites on the Env trimer.

The tier of a virus is important because this defines the sensitivity to antibody neutralisation (50), and has been associated with the conformational state of the Env trimer due to differences in epitope exposure (62, 63), as illustrated in **Figure 1**. For example, viruses that are more neutralisation resistant have a higher proportion of Env in a closed conformation. Yet viruses with high sensitivity to antibody neutralisation, such as laboratory-adapted viruses, are referred to as tier 1A/B viruses and exhibit a predominantly open/intermediate conformation respectively. Most primary isolates however are classed as tier 2 viruses that have moderate sensitivity to neutralisation, while tier 3 viruses have low sensitivity and are therefore the most resistant to neutralisation. Consequently, a panel containing a high proportion of tier 1 viruses would likely make an antibody appear to have a broader neutralisation capacity than if tested against a panel containing a high proportion of tier 2/3 viruses.

The clade of the virus is also an important factor to consider, although there is limited data on clade-based differences in neutralisation sensitivity (64). However, it has been shown in serum studies that neutralising activity is generally higher when the clade of the PV matches that of the individual’s own virus (45, 50, 65). Conflicting evidence suggests that whilst this is the case for plasma from clade C infections, it is not for clade B plasma (64, 66). The effect of virus clade on the neutralising response has also been observed with mAbs produced during natural infection or elicited by vaccination, where viruses from the same clade that stimulated the response were preferentially neutralised (67). Moreover, it has been identified that structural features characteristic of viral clades such as the presence or absence of specific residues can affect the sensitivity of neutralisation by bnAbs, but this is highly dependent on the epitope targeted (68). Therefore, a panel of viruses originating from only a single clade or circulating recombinant form (CRF) could arguably bias the neutralisation breadth achieved by the antibody tested. On the other hand, it might be informative to know if an antibody can neutralise heterologous viruses within a clade, as this level of cross-reactivity could be of benefit within certain communities. Whilst it is well-known that virus clades and CRFs are predominately found in specific geographic regions (69), it is necessary to take into account that there is often more than one clade or CRF within each region and that distributions are dynamic (70). For instance, analysis of regional distribution across Africa identified that whilst the south was almost exclusively clade C, the east was predominantly clade A, C or D yet in west and central Africa all clades and many CRFs were present, with proportions changing over time (71). Considering that Sub-Saharan Africa has the highest cases of HIV-1 in the world, this is particularly relevant and thus suggests that it would be appropriate to evaluate neutralisation breadth using viruses that reflect the diversity of HIV-1 clades/CRFs and not use virus panels that are specific for a single clade/CRF.

## Standard Virus Panels for Assessing Neutralisation Breadth

The establishment of standard PV panels that take the viral tier and clade into account has provided a consistent way to screen for broadly neutralising antibody responses and also offers a way to compare antibody neutralisation breadth. The 6 PV standard panel and 12 PV global panel consist almost entirely of tier 2 viruses from multiple clades that were shown to be representative of larger panels of viruses from the global epidemic (45, 72), thus allowing the breadth of an antibody to be assessed using only a small number of viruses. Another widely used standard panel is the 118 multi-clade PV panel which is comprised primarily of the 109 virus panel (50) and represents diverse isolates of HIV-1 from different regions of the world. It should be noted that viruses from clade F, H, J and K are not present in this panel, however even when these are combined they result in <1% of infections worldwide (71).

Even with these standard virus panels available it is clear that not all reported bnAbs have been tested against them and that the panels used to define neutralisation breadth by HIV researchers continue to vary (**Supplementary Table 1**). However, it is apparent that one virus panel has been used more frequently than others to assess bnAbs, and that is the 118 multi-clade panel. This is arguably the most appropriate standard panel for comparing bnAb neutralisation profiles as it contains more viruses than the 6 or 12 PV panels and also utilises viruses from all major clades, both of which increase confidence in the breadth achieved. Although panels containing a larger number of viruses have also been used to characterise the neutralisation profile of individual bnAbs (**Supplementary Table 1**), these are not standard PV panels that are currently recognised on the LANL HIV database CATNAP tool (24) and have not been as widely used. For these reasons the breadth and potency of neutralisation by bnAbs against the 118 multi-clade panel was chosen for analysis in this review. In total it was feasible to include 41 bnAbs for direct comparison and this has been highlighted in **Table 1**. Regrettably nAbs with limited breadth such as 447-52D and b6 (77), that are not referred to as bnAbs were not included in the comparison because these have not been tested against the 118 PV panel.

**Table 1:** Comparison of bnAb breadth against the 118 multi-clade standard PV panel.

HIV bnAb	Neutralisation breadth	Viruses tested	Potency (µg/ml)	Clades/CRFs neutralised	Reference
N49P7	100%	117	0.44	15	(30)
10E8	98%	118	0.36	14	(24)
4E10*	98%	118	1.81	15	(24)
1-18	97%	116	0.05	15	(73)
12A12	93%	117	0.22	15	(24)
LN01	92%	118	0.96	15	(74)
VRC01	91%	118	0.38	15	(24)
3BNC117	89%	118	0.12	15	(24)
PG9	87%	118	0.15	14	(24)
NIH45-46	86%	117	0.11	15	(24)
VRC13	86%	113	0.27	14	(24)
VRC-CH31	84%	115	0.32	15	(24)
PG16	83%	118	0.08	15	(24)
PGDM1400	83%	118	0.02	15	(24)
PGV04	81%	116	0.32	15	(24)
PGT145	78%	118	0.13	15	(24)
PGT151	73%	118	0.04	15	(24)
1B2530	72%	113	3.62	14	(24)
PGT128	68%	118	0.06	13	(24)
8ANC195	68%	118	1.23	15	(24)
CH103	67%	113	2.28	14	(24)
PGT121	66%	118	0.07	13	(24)
10-1074	63%	118	0.06	13	(24)
SF12	63%	118	0.21	10	(75)
PGT130	61%	117	0.20	14	(24)
BG18	61%	116	0.03	14	(24)
2F5*	58%	118	2.83	13	(24)
VRC26.08	57%	116	0.02	13	(24)
CH01	54%	115	1.38	12	(24)
VRC03	53%	115	0.80	14	(24)
35O22	51%	118	0.26	10	(24)
IOMA	49%	116	2.33	12	(24)
PCDN-33A	46%	113	0.50	11	(24)
b12*	44%	118	4.32	10	(24)
HJ16	38%	118	1.17	12	(24)
M4008_N1	36%	115	0.95	12	(76)
BG1	35%	116	0.61	11	(24)
PGT135	33%	118	0.61	13	(24)
179NC75	31%	116	0.16	10	(24)
VRC-PG05	31%	113	2.33	8	(24)
2G12*	21%	118	3.75	7	(24)

The neutralisation breadth (percentage of viruses neutralised) and the number of clades/CRFs neutralised in the 118 PV panel was captured from the antibody isolation paper or LANL HIV CATNAP tool (24), bnAbs with data for >95% of PVs in the 118 PV panel were included. Potency is given as the geometric mean IC_50_ of viruses neutralised. The total number of virus clades/CRFs in the 118 PV panel is 15, categorised according to the LANL HIV CATNAP tool. *First generation bnAbs isolated prior to 2009 are marked by an asterisk. Higher neutralisation breadth, number of viruses tested and potency are indicated by a darker shade of green, yellow and red respectively.

**Figure 2:**
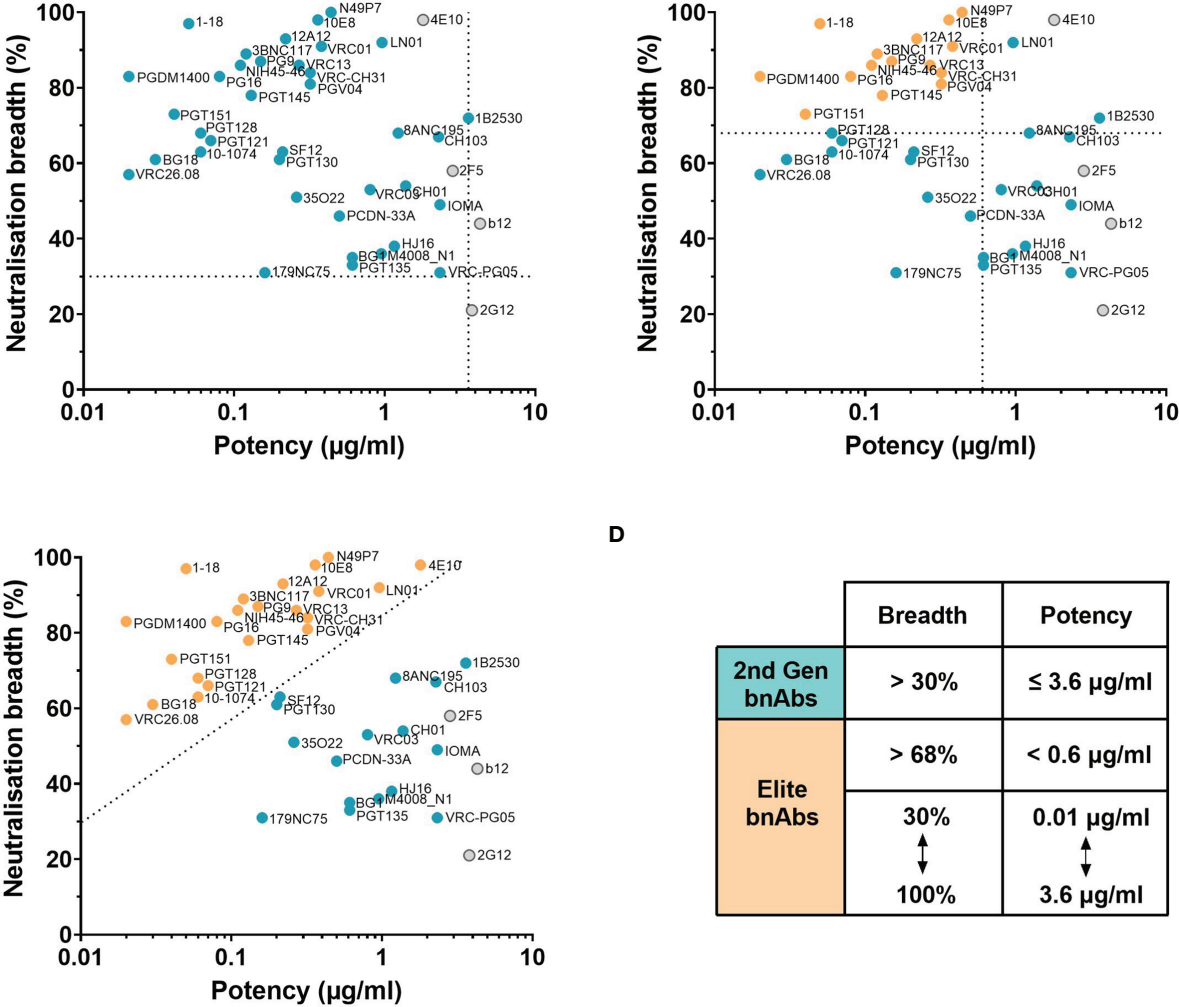
Neutralisation breadth and potency of HIV bnAbs against the 118 multi-clade PV panel. **(A–C)** First generation bnAbs isolated prior to 2009 are shown in grey and second generation bnAbs isolated after 2009 are shown in blue. **(A)** Dashed lines at 30% neutralisation breadth and potency (geometric mean IC_50_) of 3.6 µg/ml define the minimum bnAb thresholds for second generation bnAbs (blue circles). **(B)** Dashed lines at 68% neutralisation breadth and potency (geometric mean IC_50_) of 0.6 µg/ml define elite bnAbs (orange circles). **(C)** The diagonal dashed line ranging from 30% to 100% neutralisation breadth and 0.01 µg/ml to 3.6 µg/ml potency (geometric mean IC_50_) compensates lower neutralisation breadth with lower potency to define elite bnAbs (orange circles). **(D)** Summary of the criteria that categorises second generation (2^nd^ Gen) bnAbs in **(A)** and elite bnAbs in **(B, C)**.

Evaluation of bnAbs based on their neutralisation of the 118 multi-clade PV panel (**Table 1**) revealed that a minimum of 7 out of the 15 clades/CRFs in the panel are neutralised by all bnAbs. This is similar to the criterion for broad neutralisation by serum, in which elite activity is defined by the ability to neutralise a minimum of 4 clades/CRFs (45). In addition, the neutralisation breadth of bnAbs ranges from 21-100% with a geometric mean IC_50_ of 0.02-4.3 µg/ml (**Table 1**). However by taking only second generation bnAbs from **Table 1** into consideration, the thresholds of >30% neutralisation breadth and potency ≤3.6 µg/ml against the 118 PV panel (**Figure 2A**) could be used to define the minimum criterion a bnAb needs to meet. Furthermore, second generation bnAbs had an average neutralisation capacity of 68% breadth with a geometric mean IC_50_ of 0.6 µg/ml. Elite bnAbs with above average neutralisation breadth and potency could therefore be categorised as having >68% neutralisation breadth and potency of <0.6 µg/ml against the 118 PV panel (**Figure 2B**). However, this could be too strict of a cut off as 10-1074 does not fall into the category of an ‘elite’ bnAb, yet has been tested in human clinical trials and was capable of delaying viral rebound (78). Therefore it may be appropriate to instead consider that more potent bnAbs could compromise for lower breadth of neutralisation, and vice versa, as demonstrated in **Figure 2C**. The different thresholds for defining HIV-1 bnAbs based on neutralisation breadth and potency have been summarised in **Figure 2D**.

## Therapeutic Potential of Broadly Neutralising Antibodies

Analysing bnAbs based on their ability to neutralise the 118 multi-clade PV panel offers an unbiased way to compare antibody breadth and potency that could be a vital tool to identify the ‘best’ bnAbs, which are desirable for therapeutic use. However there may be instances where neutralisation breadth within a specific clade may be more beneficial than breadth against multiple clades. Nevertheless, it is hard to determine exactly how broad and potent a bnAb needs to be for successful prevention or suppression of HIV-1, although logically the closer to 100% coverage and the lower the potency the better. It is also worth considering that whilst PVs are a vital tool to characterise antibody activity *in vitro* these are not circulating viruses, and it has been shown that primary isolates derived from PBMCs can be less sensitive to bnAbs (79). In a recent press release the highly anticipated results of Phase 2b trials from the Antibody-Mediated Prevention (AMP) studies revealed that administration of the bnAb VRC01 was 75% effective at preventing acquisition of HIV strains susceptible to VRC01, however in the regions where this trial was conducted only 30% of circulating strains were VRC01-sensitive (80). This demonstrates the potential that bnAbs could have in providing protection against HIV in humans, but also highlights the prevalence of circulating strains with bnAb resistance. The use of bnAbs for the suppression of HIV-1 during interruption of antiretroviral therapy is also of high interest. In human clinical trials, administration of single bnAbs have shown that two doses of 3BNC117 suppressed viral rebound for an average of 6.7 weeks and three doses of VRC01 supressed viral rebound for a median of 4 weeks (81, 82). Although these bnAbs were evaluated in different trials the results suggest that 3BNC117 is more effective than VRC01. Interestingly, the neutralisation breadth of these two CD4bs bnAbs is similar, yet 3BNC117 is more potent than VRC01 (**Table 1**), indicating that bnAb potency is likely associated with the length of viral suppression. Findings from passive transfer studies in non-human primate models have led to the proposal that only the most potent bnAbs, with a geometric mean IC_50_ ≤0.1 µg/ml, would be capable of providing protection as viral coverage reduces with lower concentrations (47). Consistent with this, a highly potent CD4bs bnAb (1-18) with a geometric mean IC_50_ of 0.05 µg/ml was able to maintain viral suppression in HIV-1 infected humanised mice, whereas viral rebound occurred with less potent and broad CD4bs bnAbs (3BNC117 and VRC01) (73). Although these preliminary findings imply that a single bnAb could maintain suppression if broad and potent enough, this has not yet been tested in humans and there may still be risk of viral escape. The most successful human clinical trial documented so far instead used a combination of bnAbs (3BNC117 and 10-1074) targeting different sites on the HIV-1 Env and was able to maintain viral suppression for a median of 21 weeks (23). This approach is similar to the switch from monotherapy to combination antiretroviral therapy, where the administration of multiple drugs with different inhibitory mechanisms is more effective at reducing viral load and diminishing the development of drug resistance (83). In the case of bnAb therapeutics, this combination strategy or use of bi-specifics could be implemented to help prevent the emergence of HIV-1 variants capable of neutralisation escape (84, 85). Ongoing trials with modified bnAbs, bi-specific bnAbs or novel bnAb combinations [reviewed in (86–88)] will hopefully shed more light on the requirements to achieve durable as opposed to transient suppression of HIV-1. Finally, an alternative to passively transferred bnAbs could be to instead engineer B cells to express bnAbs. This approach arguably has an advantage in that a durable response could be maintained through memory cell formation and that bnAbs could undergo SHM in response to viral escape (89), although this requires further investigation.

## Can We Define HIV bnAbs by Comparing Their Levels of SHM?

During affinity maturation the introduction of SHM into immunoglobulin genes by activation-induced cytidine deaminase (AID) is random and only B cells with improved antigen binding are selected to receive signals for survival (90). Thus, rounds of mutation and selection in germinal centres generate antibodies with increased levels of SHM and higher affinity for antigen. Antigen-experienced IgG antibodies from healthy adults have been shown to possess on average 7% mutation in the variable heavy chain (VH) (91). Interestingly HIV-1 Env specific antibodies from infected individuals (reactive to gp140) have been found to have a significantly higher number of VH mutations than non-reactive antibodies, which was proposed to be a result of chronic infection (92). In agreement with this, antibodies from chronic but not acute infections in general have been found to have higher SHM (93). It has also been found that HIV bnAbs have even higher levels of mutation compared to HIV antibodies with only limited neutralisation capacity (94), and germline reversion of bnAbs to unmutated common ancestors has suggested that SHM acquired during development is essential for neutralising activity (74, 94). Longitudinal studies have also indicated that antibodies accumulate mutations in response to emerging HIV-1 Env variants and that this results in increased neutralisation breadth over time (29, 32, 33, 95). However, there are exceptions where higher VH mutation doesn’t improve breadth for antibodies from the same lineage (e.g. PGT128 vs PGT130 and PG9 vs PG16 in **Supplementary Table 1**) (46, 47). It is plausible that although antibodies from the same lineage have a common ancestor these may have diverged early during development and mutated separately, therefore the mutations acquired are likely to differ and thus affect antigen binding and breadth of neutralisation. Additionally nucleotide mutations can be silent and not impact antibody neutralisation. For example it has been suggested from the analysis of minimally mutated VRC01 that some of the mutated residues might not be required for breadth (96).

**Figure 3:**
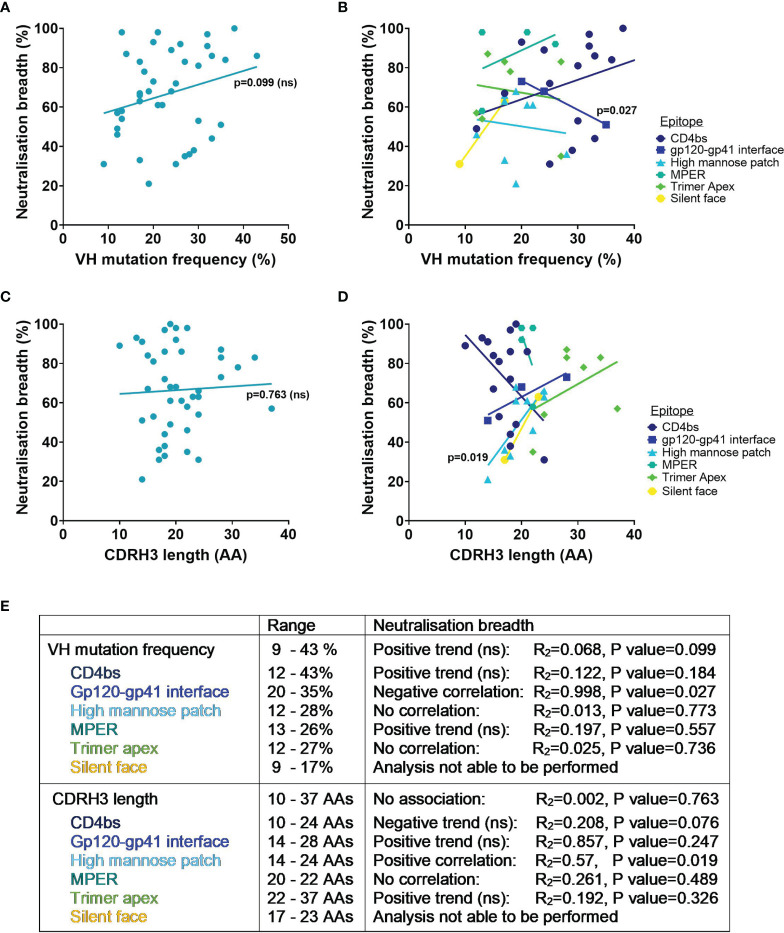
VH mutation frequency, CDRH3 length and neutralisation breadth of HIV bnAbs against the 118 multi-clade PV panel. Mutation frequency was determined from VH nucleotide sequences. Correlations were determined by linear regression analysis, with *p*<0.05. **(A)** VH mutation frequency and neutralisation breadth of bnAbs had no association (*p=*0.099). **(B)** VH mutation frequency and neutralisation breadth of bnAbs grouped by epitope was associated for gp120-gp41 interface bnAbs (*p*=0.027). **(C)** CDRH3 length and neutralisation breadth of bnAbs had no association (*p*=0.763). **(D)** CDRH3 length and neutralisation breadth of bnAbs grouped by epitope was associated for high mannose patch bnAbs (*p*=0.019). **(E)** Summary of VH mutation frequency and CDRH3 length associations with neutralisation breadth. ns, Not significant.

Whilst it is known that favourable mutations are selected during affinity maturation as they enhance binding to the specific antigen, determining the effect of SHM on the breadth of an antibody against heterologous viruses is not so simple. To try and address this, the VH mutation frequency and percentage of viruses neutralised have been compared here for bnAbs tested against the 118 PV standard panel. Interestingly, the neutralisation breadth of all HIV bnAbs did not have a significant correlation with higher SHM in the VH (p=0.099, **Figure 3A**), nor when they were grouped by the epitopes targeted (**Figure 3B**). Instead only a negative correlation between VH mutation and neutralisation breadth for gp120-gp41 interface bnAbs was observed (p=0.027, **Figure 3B**). Nevertheless, this did highlight that a VH mutation frequency of ≥ 9% was exhibited by all bnAbs (**Figure 3E**). Although this might be the case for bnAbs from adults, it has been demonstrated that HIV-1 infected infants can produce bnAbs without higher levels of SHM (27, 97). Indeed infant-derived bnAbs BF520.1 and AIIMS-P01 that target the Env high mannose patch have only a 7% VH mutation and both achieved 58% breadth against the 12 global PV panel (24, 27, 97). For comparison the adult-derived bnAb VRC29.03, targeting the same epitope, also achieved 58% breadth against this panel but has more than double the VH mutation (**Supplementary Table 1**). Unfortunately it is not possible to compare the breadth of VRC29.03 to the breadth of other bnAbs in **Table 1** because this bnAb has not been tested against the same panel of PVs. The humoral response of infants could have an advantage over adults by developing in the presence of maternal HIV-1 specific antibodies that may enhance the *de novo* response (16). This would be unsurprising considering that neutralisation breadth can be driven by the cooperation of antibody lineages (98–100). Conversely far fewer bnAbs from infants have been isolated than adults and so the neutralisation breadth of BF520.1 and AIIMS-P01 achieved without extreme SHM may be outliers. Furthermore, the immune system of infants differs substantially from adults [as reviewed in (101)], which raises the question whether their antibodies can be fairly compared to those of adults.

Another aspect to consider is that antibody variable regions are comprised of CDRs and framework regions (FWRs) with SHM typically accumulating in CDR loops for improved interaction with antigen. Mutations are less tolerated in the FWRs, which support the three CDR loops per antibody chain, as changes in these regions are more likely to adversely affect the overall structure of the antibody. However, unlike most antibodies some HIV-1 bnAbs have a high number of FWR mutations that appear to be essential for their broad and potent neutralisation, by increasing flexibility of binding or through contacts with the antigen (94, 102). On the other hand, analysis of engineered variants for two bnAbs (VRC01 and 10E8) has instead implied that broad neutralisation can be achieved even when framework regions are reverted considerably (103). This suggests that only minimal framework mutations are required for these particular bnAbs and that additional mutations serve merely to improve the neutralisation potency.

## Can We Define HIV bnAbs by Comparing Their CDRH3 Length?

The length of the CDR loops can also affect antigen binding, and the heavy chain CDR3 (CDRH3) in particular is important for determining antigen specificity because it contains the most sequence diversity due to VDJ gene recombination in the pre-B cell (104). During development B cells undergo selection processes that leads to a relatively low frequency of B cell clones with long (>20 AAs) CDRH3 sequences (105–107), meaning these are infrequent genetic features in antibody repertoires, often being removed due to being auto-reactive (108). Remarkably many HIV-1 bnAbs have long CDRH3 loops that exceed the most frequent length of 14 amino acids and mean length of 15-16 amino acids (107, 109, 110). It is perhaps unsurprising that this genetic feature is present in HIV bnAbs given that longer CDRH3s have more opportunity for sequence variation and the potential to access recessed epitopes on antigens. Curiously CDRH3 sequences as long as that of the bnAb PG9 (28 AA) were revealed to be present in B cell repertoires from HIV-1 naïve individuals (111), suggesting that this feature, while relatively rare, is not unique to HIV bnAbs.

Considering that other CDR loops on the antibody heavy and light chains also contribute to antigen binding it is unlikely that the CDRH3 alone accounts for the neutralisation breadth of bnAbs. In agreement with this, there was no association of neutralisation breadth for bnAbs from **Table 1** with the length of their CDRH3 (p=0.763, **Figure 3C**). Yet when bnAbs were grouped by the epitopes targeted (**Figure 3D**) this revealed that a longer CDRH3 correlated with increased neutralisation breadth for bnAbs targeting the high mannose patch (p=0.019). Structural analysis has demonstrated that bnAbs against this epitope require extended CDRH3 loops to penetrate through the glycan shield and contact the more conserved envelope protein residues below (32, 112), offering a possible explanation for the association of CDRH3 length with breadth. Trimer apex bnAbs all had CDRH3 lengths of ≥22 AAs, highlighting the known requirement for a long CDRH3 to be able to access this epitope (113). However the trend between increased CDRH3 length with increased neutralisation breadth of apex bnAbs was not significant (**Figures 3D, E**). For the MPER epitope a long CDRH3 of 20-22 AAs appeared to be characteristic for all bnAbs but showed no correlation with breadth (**Figures 3D, E**). And while an increase in neutralisation breadth was observed for bnAbs with longer CDRH3s that target the gp120-gp41 interface and silent face, only a limited number of bnAbs were able to be included in this analysis and the association was not found to be significant (**Figures 3D, E**). In contrast for CD4bs bnAbs the length of the CDRH3 had a negative trend with neutralisation breadth, although this was not significant (p=0.076, **Figure 3E**). Considering that the majority of CD4bs bnAbs are VH gene restricted and bind predominantly *via* their CDRH2 (114, 115), rather than their CDRH3 loop, this is perhaps unsurprising.

Overall the level of SHM and the CDRH3 length of bnAbs required for neutralisation breadth (against the 118 PV panel) differ depending on the site being targeted on the HIV-1 Env and their mode of binding. Although HIV bnAbs have a high VH mutation and/or a long CDRH3, an increase in VH mutation did not correlate with increased breadth for any bnAbs and only an increase in CDRH3 length correlated with increased neutralisation breadth for those targeting the high mannose patch.

## Can We Define Breadth by Comparing Epitopes of bnAbs With Non-bnAbs?

The limited number of properly folded, functional Env trimers present on the surface of each virion is thought to be one of the many ways HIV is able to evade the immune system (1). Unprocessed Env protein that is not cleaved into gp120-gp41 heterodimers is non-functional, and as a result exposes non-neutralising epitopes (116). In addition, the instability of the Env trimer results in various forms such as non-functional gp41 stumps (depleted of gp120) and gp120-gp41 monomers being presented on the virion surface. These forms of Env are widely thought to act as decoys to the immune system by displaying epitopes (cluster I and II) on gp41 that are usually occluded by the trimer and so antibodies elicited to these sites that are able to bind but not neutralise HIV (117). Moreover, the shedding of gp120 from Env leads to the presence of circulating gp120 monomers that expose immunodominant sites and elicit non-neutralising antibodies (non-nAbs). Whilst some non-nAbs directed towards gp120 bind overlapping epitopes to nAbs, such as those in the CD4bs, V2 and V3 loop, their means of approach are not possible when gp120 is packed into a functional trimer (118–122). In agreement with this, immunisation studies using subunits of Env have had little success in eliciting desirable nAbs in comparison to native-like trimers [as reviewed in (123)]. It is therefore relatively easy to identify non-nAbs based on epitope alone due to their manner of binding and inability to target functional Env trimers, however distinguishing between nAbs and bnAbs based on their epitope is somewhat more complicated.

## Neutralising Antibodies Targeting the CD4bs

HIV initially requires interactions with CD4 on the host cell to gain entry, this is mediated by the CD4 receptor binding site (CD4bs) on the Env trimer and is therefore a functionally conserved region and a site of vulnerability. The CD4bs is situated on the gp120 subunit in a recessed hydrophobic pocket at the interface of the outer and inner domains (124), and is less accessible on the Env trimer compared to monomeric gp120 (125). Despite this, neutralising antibodies can be elicited against the CD4bs, although access has been demonstrated to be dependent on the angle of approach (126, 127). This helps to explain why some CD4bs antibodies are only capable of neutralising tier 1 viruses that exhibit a more open Env trimer with fewer conformational constraints than tier 2/3 viruses (62, 128). CD4bs bnAbs however can neutralise tier 2/3 viruses through distinct approaches of binding, predominantly using their CDRH2 (for antibodies with VH1-2 or VH1-46) or their CDRH3.

The majority of CD4bs nAbs and bnAbs contact the highly conserved residue D368 on the CD4 binding loop (115), with only a few exceptions that instead are glycan dependant (129–131). As glycans do not substantially mask the CD4bs they are not often incorporated into CD4bs antibody epitopes. Nevertheless, glycans surrounding the binding site (e.g. N276 and N462) limit antibody access but can be accommodated or even bound by bnAbs with short, compact light chain CDR (CDRL) loops to prevent clashing which likely contributes to their neutralisation breadth (40, 130, 132). In addition, structural analysis of the VH1-2 restricted, VRC01-class bnAbs identified that a short or flexible CDRL1 is necessary to avoid steric clashes with Loop D (114). Recognition of the CD4bs by bnAbs has also been found to differ from non-bnAbs in clustering analysis due to their ability to contact residues further into the binding site (133). Similarly, longitudinal analysis of the CH235 antibody lineage revealed that progression towards the bnAb CH235.12 was driven by SHM, resulting in more specific contacts within the CD4 binding loop yet reduced contact with variable regions in close proximity such as the V5 loop (95). Indeed in a different study the limited neutralisation breadth displayed by the CD4bs nAb CAP257-RH1 was a consequence of its binding angle, which was incompatible with glycosylated V5 loops (129). Furthermore the most effective CD4bs bnAbs isolated to date (N6 that demonstrated 98% neutralisation breadth against a panel of 181 PVs and N49P7 with 100% neutralisation breadth against 117 of the 118 PV panel) have acquired extensive mutations to bury their CDRH2 into gp120 and engage conserved residues in the CD4 binding loop (30, 134). This mimics CD4 binding, which is common of many bnAbs in the VRC01-class and contributes to their neutralisation breadth (114, 135, 136).

## Neutralising Antibodies Targeting the V3

Another conserved region of Env is the V3 loop, which consists of three main structural regions: the base and the tip (also referred to as a crown), which are involved in co-receptor binding, and the more variable stem (137). However the V3 loop is buried beneath the V1/2 domain and not accessible until conformational changes cause the trimer to open upon CD4 engagement (138, 139). Most antibodies that target the V3 loop are therefore only capable of neutralising tier 1 viruses with a predominantly open conformation (similar to the CD4-bound state), this along with the high sequence variability of the V3 stem often leads to these antibodies being strain-specific (128). Conversely it has been demonstrated that V3 specific nAbs that target the V3 tip/crown, such as 447-52D, have the potential to neutralise some tier 2 viruses, although only weak/incomplete neutralisation has been observed which implies poor or transient accessibility to the epitope (67, 140). Recent analysis of strain-specific neutralising responses (from immunised rabbits) revealed an overlapping footprint at the base of the V3 with that of high mannose patch bnAbs, yet the specific epitope of these autologous antibodies and binding mechanism differed by contacting peptide residues in the V1 loop and not V3 glycans (141). In agreement with this, a longitudinal study has suggested that affinity for V3 glycans acquired during antibody evolution subsequently widens the breadth of neutralisation (32). Interestingly, V3 antibody neutralisation also correlates with the Env V1 loop length whereby breadth is achieved as longer loops are accommodated, although this often reduces antibody potency (33). These findings correspond with the fact that the conserved (^324^GDIR^327^) linear sequence at the base of the V3 loop is masked by the V1/V2 domain as well as the N-linked glycan supersite (N295, N301, N332, N339, N385 and N392) in the closed (pre-fusion) conformation of Env, typical of most circulating tier 2 viruses (142, 143). It has been demonstrated that bnAbs to this epitope are able to incorporate glycans into their epitope, in particular the N332 glycan, as well as use long CDR loops to reach past them to contact the conserved peptide residues (112, 144). The only exception being bnAb 2G12 which relies solely on contacting glycans (145). Often multiple V3 glycans or glycans close to this site, such as those protruding from the V1 loop, can also be utilised by high mannose patch bnAbs and consequently allows a degree of flexibility in their epitope and different angles of approach (33, 146–150). The breadth of neutralisation achieved by bnAbs against the high mannose patch is therefore aided by the ability to accommodate different glycans, due to changes in N-linked glycosylation sites often being a way that HIV is able to escape from neutralisation (10, 146).

## Neutralising Antibodies Targeting the V1/V2

Finally, the V1/V2 domain is another site on the gp120 subunit of Env that is targeted by the humoral response, and although this site itself is not directly involved in viral entry the V1/V2 loops are necessary to shield the region involved in co-receptor binding until it is required (151, 152). Neutralising antibodies directed to the hypervariable V1/V2 domain can therefore prevent opening of the trimer and exposure of the host cell co-receptor required for viral engagement. In the native Env trimer, the V1/V2 domain is located at the apex and is comprised of a five-stranded β-barrel, with conserved residues being located in the strands and more variable residues in the loops connecting these strands (153). However, it has also been revealed that residues in the V2 domain can adopt a coil or helical conformation in CD4-bound open trimers that result in the exposure of (short linear peptide) epitopes in tier 1A viruses that can be bound by nAbs (122, 154). Analysis of ‘tier 1A’ nAbs have revealed similarities with bnAbs in the residues contacted on Env, such as those at position 160 and 168-171 in the positively-charged V2 site (113, 122, 155, 156), however the secondary structure of V2 in different Env conformations results in distinct epitopes. Furthermore, bnAbs targeting the trimer apex have been shown to preferentially recognise the quaternary structure of Env (exhibiting a β-conformation of V2) (157), and thus bind intermediate and/or closed conformations of the Env trimer exhibited by tier 1B and tier 2/3 viruses, rather than open conformations characteristic of tier 1A viruses (62, 158). Antibodies that exhibit strain-specific neutralisation have also been identified against the Env quaternary structure, however in the case of the nAb 2909 this can be explained by the reliance on K160 for neutralisation rather than the highly conserved N160 and thus has limited breadth (159, 160). In contrast bnAbs are dependent on glycans at the trimer apex, interacting with or requiring the presence of the N156 and N160 glycan protruding from the V2 strand B, with the latter being essential in bnAb epitopes as demonstrated by mutant viruses lacking N160 becoming partially or completely resistant to neutralisation (9, 113, 161–165). These glycans mask both the hole at the trimer apex and the positively-charged lysine-rich site on the V2 strand C which form epitopes that can be accessed by bnAbs using a long, negatively charged CDRH3 often containing a YYD motif with sulphated tyrosines (166). An exception to this being VRC38.01 which has a CDRH3 length of 16 AAs that may restrict its neutralisation breadth to 30% against the 208 PV panel, **Supplementary Table 1** (163). Furthermore V2 nAbs with more limited neutralisation capacity have been found to possess shorter CDRH3s than most apex bnAbs, and are often restricted by viruses with long V2 loops (46, 167). However, simply possessing a long CDRH3 is not enough to confer breadth, as demonstrated by the CAP256 antibody lineages, this loop must also protrude out and away from the rest of the antibody with an appropriate orientation (164). Nevertheless, a different binding approach has recently been displayed by bnAb BG1, where a protruding CDRH2 rather than CDRH3 contacts the protein residues beneath glycans, however these interactions appear to be more easily disrupted by changes in Env residues and may account for its lower neutralisation breadth compared to other apex bnAbs (9).

Overall, although the binding footprint of HIV bnAbs and nAbs are often similar, the angle of approach or specific residues contacted differ to enable bnAbs to neutralise a broad spectrum of viruses. In addition, bnAbs are able to accommodate glycans or even incorporate them into their epitope through the acquisition of beneficial mutations to allow a degree of flexibility in their binding. However even bnAbs against the same region on Env can vary in the residues that they engage, and whilst the epitopes of bnAbs have been well characterised the epitopes of nAbs have been studied less, thus making it challenging to predict the neutralisation breadth of an antibody based on epitope alone.

## Summary

HIV bnAbs are of particular interest in HIV-1 research due to many studies demonstrating their potential to provide protection, and thus have been extensively studied. However, despite the vast amount of knowledge acquired about these antibodies there is no set of criteria to determine or define what it means to be a bnAb. This question has been addressed here by comparing bnAbs based on their neutralisation breadth and potency, their mutational frequencies and CDRH3 lengths and the epitopes targeted. The majority of bnAbs previously tested against the 118 multi-clade PV panel can neutralise ≥7 clades/CRFs and achieve >30% neutralisation breadth with a geomean IC_50_ ≤3.6 µg/ml. In addition, bnAbs have a wide range of VH mutation (9-43%) and CDRH3 length (10-37AAs) but overall these features did not correlate with an increase in neutralisation breadth. Finally, it is hard to distinguish bnAbs solely on the epitope targeted because their footprints can, to a degree, overlap with those of nAbs.

In conclusion, while some traits are common to many bnAbs (high SHM, long CDRH3s or epitope specificity) it would not have been possible to identify current HIV-1 bnAbs based on these alone. Without prior knowledge of their neutralisation breadth these bnAbs are not distinctive enough from those of nAbs with limited neutralisation breadth. Regrettably this prevents new bnAbs from being discovered directly from repertoire sequencing (168) or from antibody binding footprints determined by competition binding experiments or epitope mapping of polyclonal serum (169), posing a challenge for defining immune profiles associated with bnAb development. Therefore individual bnAbs must continue to be characterised initially by functional screening to identify neutralisation breadth comparable to those that have already been isolated.

## Author Contributions

SAG performed the literature searches, data collection and wrote the manuscript. LEM revised the final version of the manuscript. All authors contributed to the article and approved the submitted version.

## Funding

LEM is supported by an MRC Career Development Award MR/R008698/1 and receives funding from the European Research Council (ERC) under the European Union’s Horizon 2020 research and innovation programme (Grant Agreement No. 757601).

## Conflict of Interest

The authors declare that the research was conducted in the absence of any commercial or financial relationships that could be construed as a potential conflict of interest.

## Publisher’s Note

All claims expressed in this article are solely those of the authors and do not necessarily represent those of their affiliated organizations, or those of the publisher, the editors and the reviewers. Any product that may be evaluated in this article, or claim that may be made by its manufacturer, is not guaranteed or endorsed by the publisher.
