# Supplementary material for: To bnAb or Not to bnAb: Defining Broadly Neutralising Antibodies Against HIV-1
Source: Front Immunol. 2021 Oct 19;12:708227. doi: 10.3389/fimmu.2021.708227 (PMC8560739; doi:10.3389/fimmu.2021.708227)
Supplement: Supplementary file 1 [file Table_1.docx]

**Supplementary Table 1** **Genetic features and neutralisation profiles of HIV-1 bnAbs.** Mutation frequency was determined from nucleotide (or amino acid*) sequence of the variable (V) regions for heavy and light chain antibody genes. For the neutralisation breadth, an inhibitory concentration that reduces viral infectivity by 50% (IC_50_) cut-off of 50 µg/ml was used to determine the percentage of viruses neutralised (coverage) in a given PV panel, with the potency indicating the geometric mean IC_50_ of viruses neutralised. Higher coverage, number of viruses tested and potency are indicated by a darker shade of colour. The confidence interval of neutralisation breadth reflects the percentage of viruses neutralised in the 6 PV standard panel (A), 12 PV global panel (B) and 118 multi-clade PV panel (C) by bnAbs, data was captured from the LANL CATNAP tool (1) or bnAb isolation paper. Only bnAbs tested against >95% of PVs in the 118 PV panel were included. Any data that could not be determined (nd) is stated.

| **Epitope** | **bnAb** | **Viral clade** | **Year** | **V gene usage (& mutation frequency)** | | **CDR3 length (AA)** | | **Reference** | **Author defined neutralisation breadth** | | | **Neutralisation breadth confidence interval (%)** | |
| --- | --- | --- | --- | --- | --- | --- | --- | --- | --- | --- | --- | --- | --- |
|  |  |  |  |  |  |  |  |  | **Coverage  (IC_50_ <50 µg/ml)** | **Number of viruses tested** | **Potency (µg/ml)** |  |  |
| CD4 bs | b12 | B | 1994 | VH1-3 (33%) | VK3-20 (19%)* | H:18 | L:9 | (2, 3) | **75%** | 16 | 2.07 | 17-67% | ABC |
|  | HJ16 | C | 2010 | VH3-30 (29%) | VK4-1 (32%) | H:18 | L:8 | (4, 5) | **39%** | 46 | 1.6 | 38-58% | BC |
|  | VRC01 | B | 2010 | VH1-2 (32%) | VK3-11 (17%) | H:14 | L:5 | (6) | **91%** | 190 | 0.33 | 91-92% | BC |
|  | VRC03 | B | 2010 | VH1-2 (30%) | VK3-20 (20%) | H:16 | L:5 | (6) | **57%** | 190 | 0.45 | 53% | C |
|  | 1B2530 | nd | 2011 | VH1-46 (25%) | VL1-47 (16%) | H:18 | L:11 | (7) | **72%** | 118 | 3.61 | 72% | C |
|  | PGV04 | AD | [2011](https://www.ncbi.nlm.nih.gov/pmc/articles/PMC5086270/#R4) | VH1-2 (30%) | VK3-20 (19%) | H:16 | L:5 | (8) | **76%** | 178 | 0.196 | 81% | C |
|  | NIH45-46 | B | 2011 | VH1-2 (33%) | VK3-11 (17%) | H:18 | L:5 | (7) | **86%** | 118 | 0.1 | 86% | C |
|  | 12A12 | nd | 2011 | VH1-2 (20%) | VK1-33 (14%) | H:13 | L:5 | (7) | **92%** | 118 | 0.27 | 93% | C |
|  | 3BNC117 | B | 2011 | VH1-2 (24%) | VK1-33 (16%) | H:10 | L:5 | (7) | **92%** | 118 | 0.15 | 89-92% | BC |
|  | VRC-CH31 | A | 2011 | VH1-2 (36%) | VK1-33 (18%) | H:15 | L:5 | (8) | **83%** | 80 | 0.098 | 84-92% | BC |
|  | CH103 | C | 2013 | VH4-59 (17%) | VL3-1 (11%) | H:15 | L:10 | (9) | **55%** | 196 | 4.54 | 67% | C |
|  | VRC23 | B | 2013 | VH1-2 (27%)* | VK3-15 (22%)* | H:12 | L:5 | (10) | **63%** | 178 | 2.4† |  |  |
|  | VRC-PG20 | A | 2013 | VH1-2 (24%) | VL2-14 (15%) | H:13 | L:5 | (11) | **77%** | 178 | 0.17 |  |  |
|  | VRC13 | B | 2015 | VH1-69 (43%) | VL2-14 (22%)* | H:21 | L:6 | (4) | **82%** | 175 | 0.113 | 86% | C |
|  | 179NC75 | nd | 2015 | VH3-21 (25%) | VL3-1 (18%) | H:24 | L:10 | (12) | **37%** | 120 | 0.113 | 31% | C |
|  | CH235.12 | C | 2016 | VH1-46 (26%) | VK3-15 (13%) | H:15 | L:8 | (13) | **89%** | 202 | 0.66 |  |  |
|  | N6 | nd | 2016 | VH1-2 (31%) | VK1-33 (25%) | H:15 | L:5 | (14) | **98%** | 181 | 0.04 | 100% | B |
|  | IOMA | B | 2016 | VH1-2 (12%) | VL2-23 (8%) | H:19 | L:8 | (15) | **50%** | 118 | 2.32 | 49% | C |
|  | N49P7 | nd | 2018 | VH1-2 (38%) | VL2-11 (31%) | H:19 | L:5 | (16) | **100%** | 117 | 0.44 | 100% | C |
|  | 1-18 | B | 2020 | VH1-46 (32%) | VK3-20 (21%) | H:18 | L:9 | (17) | **97%** | 119 | 0.05 | 97% | C |

**Supplementary Table 1 continued**

| **Epitope** | **bnAb** | **Viral clade** | **Year** | **V gene usage (& mutation frequency)** | | **CDR3 length (AA)** | | **Reference** | **Author defined neutralisation breadth** | | | **Neutralisation breadth confidence interval (%)** | |
| --- | --- | --- | --- | --- | --- | --- | --- | --- | --- | --- | --- | --- | --- |
|  |  |  |  |  |  |  |  |  | **Coverage  (IC_50_ <50 µg/ml)** | **Number of viruses tested** | **Potency (µg/ml)** |  |  |
| V3 / High mannose patch | 2G12 | AD | 1994 | VH3-21 (19%) | VK1-5 (13%) | H:14 | L:9 | (18, 19) | Not defined in isolation paper | | | 21-50% | ABC |
|  | PGT121 | A | [2011](https://www.ncbi.nlm.nih.gov/pmc/articles/PMC5086270/#R15) | VH4-59 (17%) | VL3-21 (18%) | H:24 | L:12 | (20) | **70%** | 162 | 0.07 | 66-87% | ABC |
|  | PGT128 | AG | 2011 | VH4-39 (19%) | VL2-8 (9%) | H:19 | L:10 | (20) | **72%** | 162 | 0.05 | 68-100% | ABC |
|  | PGT130 | AG | [2011](https://www.ncbi.nlm.nih.gov/pmc/articles/PMC5086270/#R15) | VH4-39 (22%) | VL2-8 (12%) | H:19 | L:10 | (20) | **52%** | 162 | 0.24 | 61-100% | AC |
|  | PGT135 | C | [2011](https://www.ncbi.nlm.nih.gov/pmc/articles/PMC5086270/#R15) | VH4-39 (17%) | VK3-15 (16%) | H:18 | L:9 | (20) | **33%** | 162 | 0.35 | 33-60% | AC |
|  | 10-1074 | A | 2012 | VH4-59 (17%) | VL3-21 (16%) | H:24 | L:12 | (21) | **57%** | 119 | 0.05 | 63-67% | BC |
|  | PGDM12 | nd | 2016 | VH3-11 (32%) | VK2-24 (15%) | H:21 | L:9 | (22) | **54%** | 106 | 0.18 |  |  |
|  | PGDM21 | nd | 2016 | VH4-34 (22%) | VK3-20 (15%) | H:20 | L:9 | (22) | **49%** | 106 | 0.1† |  |  |
|  | PCDN-33A | C | 2016 | VH4-34 (12%) | VK3-20 (11%) | H:22 | L:8 | (23) | **47%** | 110 | 0.53 | 46-67% | AC |
|  | VRC29.03 | B | 2016 | VH4-59 (15%) | VK3-20 (9%) | H:20 | L:9 | (24) | **29%** | 208 | 1.3 | 58% | B |
|  | VRC41.01 | nd | 2017 | VH4-39 (21%) | VK3-20 (14%) | H:21 | L:9 | (25) | **56%** | 177 | 0.78 |  |  |
|  | BG18 | B | 2017 | VH4-4 (21%) | VL3-25 (18%) | H:21 | L:11 | (26) | **64%** | 118 | 0.03 | 61% | C |
|  | DH270.6 | C | 2017 | VH1-2 (13%) | VL2-23 (8%) | H:20 | L:10 | (27) | **55%** | 207 | 1.36 | 67% | B |
|  | M4008_N1 | B | 2020 | VH1-69 (28%) | VK1-5 (24%) | H:17 | L:9 | (28) | **42%** | 120 | 0.77 | 36% | C |
|  | 438-B11 | B | 2020 | VH1-69 (25%) | VK3-20 (13%) | H:21 | L:9 | (29) | **42%** | 129 | 0.06 | 67-83% | AB |
| Trimer Apex | PG9 | A | [2009](https://www.ncbi.nlm.nih.gov/pmc/articles/PMC5086270/#R13) | VH3-33 (14%) | VL2-14 (10%) | H:28 | L:11 | (30) | **78%** | 162 | 0.28 | 83-92% | ABC |
|  | PG16 | A | [2009](https://www.ncbi.nlm.nih.gov/pmc/articles/PMC5086270/#R13) | VH3-33 (17%) | VL2-14 (14%) | H:28 | L:11 | (30) | **73%** | 162 | 0.19 | 83-92% | ABC |
|  | PGT145 | A / D | [2011](https://www.ncbi.nlm.nih.gov/pmc/articles/PMC5086270/#R15) | VH1-8 (18%) | VK2-28 (16%) | H:31 | L:9 | (20) | **78%** | 162 | 0.28 | 83-78% | AC |
|  | CH01 | A | [2011](https://www.ncbi.nlm.nih.gov/pmc/articles/PMC5086270/#R14) | VH3-20 (13%) | VK3-20 (10%) | H:24 | L:9 | (31) | [**46%**](https://www.ncbi.nlm.nih.gov/pmc/articles/PMC3196428/figure/F2/) | 91 | 0.74 | 54-75% | BC |
|  | PGDM1400 | A / D | [2014](https://www.ncbi.nlm.nih.gov/pmc/articles/PMC5086270/#R18) | VH1-8 (27%) | VK2-28 (11%) | H:34 | L:9 | (32) | **83%** | 106 | 0.003† | 67-92% | ABC |
|  | VRC26.08 | C | 2014 | VH3-30 (12%) | VL1-51 (10%) | H:37 | L:12 | (33) | **47%** | 47 | 0.11 | 50-67% | ABC |
|  | VRC26.25 | C | 2016 | VH3-30 (12%) | VL1-51 (10%) | H:36 | L:12 | (34) | **57%** | 183 | 0.002 | 83% | B |
|  | BG1 | B | 2017 | VH3-49 (27%) | VK1-39 (20%) | H:22 | nd | (26) | **37%** | 118 | 0.67 | 35% | B |
|  | VRC38.01 | B | 2017 | VH3-13 (18%) | VK2-28 (9%) | H:16 | L:10 | (35) | **30%** | 208 | 0.46 | 33-50% | AB |
|  | PCT64-35S | A | 2017 | VH3-15 (13%) | VK3-20 (7%) | H:25 | L:8 | (36) | **27%** | 109 | 0.62 | 50% | A |

**Supplementary Table 1 continued**

| **Epitope** | **bnAb** | **Viral clade** | **Year** | **V gene usage (& mutation frequency)** | | **CDR3 length (AA)** | | **Reference** | **Author defined neutralisation breadth** | | | **Neutralisation breadth confidence interval (%)** | |
| --- | --- | --- | --- | --- | --- | --- | --- | --- | --- | --- | --- | --- | --- |
|  |  |  |  |  |  |  |  |  | **Coverage  (IC_50_ <50 µg/ml)** | **Number of viruses tested** | **Potency (µg/ml)** |  |  |
| MPER | 2F5 | B | 1994 | VH2-5 (13%) | VK1-13 (11%) | H:22 | L:9 | (18, 37, 38) | Not defined in isolation paper | | | 33-58% | ABC |
|  | 4E10 | B | 1994 | VH1-69 (13%) | [VK3-20 (7%)](https://www.ncbi.nlm.nih.gov/pmc/articles/PMC4040451/) | H:20 | L:9 | (18, 39) | Not defined in isolation paper | | | 83-100% | ABC |
|  | 10E8 | B | [2012](https://www.ncbi.nlm.nih.gov/pmc/articles/PMC5086270/#R17) | VH3-15 (21%) | VL3-19 (14%) | H:22 | L:12 | (40) | **98%** | 181 | 0.22 | 98-100% | BC |
|  | DH511 -2 | C | 2017 | VH3-15 (18%) | VK1-39 (14%) | H:23 | L:11 | (41) | **99%** | 208 | 0.79 | 100% | B |
|  | LN01 | B | 2019 | VH4-39 (26%) | VK1-39 (27%)* | H:20 | L:9 | (42) | **92%** | 118 | 0.96 | 92% | C |
| gp120 -gp41 interface | 8ANC195 | B | 2011 | VH1-69 (24%) | VK1-5 (15%) | H:20 | L:9 | (7, 43) | **67%** | 118 | 1.23 | 68-83% | BC |
|  | 3BC176 | B | 2012 | VH1-2 (23%) | VL2-23 (22%)* | H:19 | L:10 | (44, 45) | **54%** | 39 | 0.97 |  |  |
|  | PGT151 | C | 2014 | VH3-30 (20%) | VK2-29 (12%) | H:28 | L:9 | (46) | **66%** | 117 | 0.022 | 73-83% | BC |
|  | 35O22 | B | [2014](https://www.ncbi.nlm.nih.gov/pmc/articles/PMC5086270/#R21) | VH1-18 (35%) | VL2-14 (24%) | H:14 | L:10 | (47) | **62%** | 181 | 0.06 | 51-58% | BC |
|  | ACS202 | B | 2016 | VH3-30 (43%) | VK1-33 (18%) | H:24 | L:9 | (48) | **45%** | 75 | 0.142† |  |  |
|  | VRC34.01 | B | [2016](https://www.ncbi.nlm.nih.gov/pmc/articles/PMC5086270/#R12) | VH1-2 (15%) | VK1-9 (10%) | H:13 | L:9 | (49) | **49%** | 208 | 0.32 | 75% | B |
| Silent face | VRC-PG05 | AD | 2018 | VH3-7 (9%) | VK4-1 (6%) | H:17 | L:8 | (50) | **27%** | 208 | 0.8 | 31% | C |
|  | SF12 | nd | 2019 | VH4-59 (17%) | VK3-20 (15%) | H:23 | L:6 | (51) | **62%** | 119 | 0.2 | 63-67% | BC |

**References**

1. Yoon H, Macke J, West AP, Jr., Foley B, Bjorkman PJ, Korber B, et al. CATNAP: a tool to compile, analyze and tally neutralizing antibody panels. Nucleic Acids Res. 2015;43(W1):W213-9.

2. Burton DR, Pyati J, Koduri R, Sharp SJ, Thornton GB, Parren PW, et al. Efficient neutralization of primary isolates of HIV-1 by a recombinant human monoclonal antibody. Science. 1994;266(5187):1024-7.

3. Zwick MB, Parren PW, Saphire EO, Church S, Wang M, Scott JK, et al. Molecular features of the broadly neutralizing immunoglobulin G1 b12 required for recognition of human immunodeficiency virus type 1 gp120. J Virol. 2003;77(10):5863-76.

4. Zhou T, Lynch RM, Chen L, Acharya P, Wu X, Doria-Rose NA, et al. Structural Repertoire of HIV-1-Neutralizing Antibodies Targeting the CD4 Supersite in 14 Donors. Cell. 2015;161(6):1280-92.

5. Corti D, Langedijk JP, Hinz A, Seaman MS, Vanzetta F, Fernandez-Rodriguez BM, et al. Analysis of memory B cell responses and isolation of novel monoclonal antibodies with neutralizing breadth from HIV-1-infected individuals. PLoS One. 2010;5(1):e8805.

6. Wu X, Yang ZY, Li Y, Hogerkorp CM, Schief WR, Seaman MS, et al. Rational design of envelope identifies broadly neutralizing human monoclonal antibodies to HIV-1. Science. 2010;329(5993):856-61.

7. Scheid JF, Mouquet H, Ueberheide B, Diskin R, Klein F, Oliveira TY, et al. Sequence and structural convergence of broad and potent HIV antibodies that mimic CD4 binding. Science. 2011;333(6049):1633-7.

8. Wu X, Zhou T, Zhu J, Zhang B, Georgiev I, Wang C, et al. Focused evolution of HIV-1 neutralizing antibodies revealed by structures and deep sequencing. Science. 2011;333(6049):1593-602.

9. Liao HX, Lynch R, Zhou T, Gao F, Alam SM, Boyd SD, et al. Co-evolution of a broadly neutralizing HIV-1 antibody and founder virus. Nature. 2013;496(7446):469-76.

10. Georgiev IS, Doria-Rose NA, Zhou T, Kwon YD, Staupe RP, Moquin S, et al. Delineating antibody recognition in polyclonal sera from patterns of HIV-1 isolate neutralization. Science. 2013;340(6133):751-6.

11. Zhou T, Zhu J, Wu X, Moquin S, Zhang B, Acharya P, et al. Multidonor analysis reveals structural elements, genetic determinants, and maturation pathway for HIV-1 neutralization by VRC01-class antibodies. Immunity. 2013;39(2):245-58.

12. Freund NT, Horwitz JA, Nogueira L, Sievers SA, Scharf L, Scheid JF, et al. A New Glycan-Dependent CD4-Binding Site Neutralizing Antibody Exerts Pressure on HIV-1 In Vivo. PLoS Pathog. 2015;11(10):e1005238.

13. Bonsignori M, Zhou T, Sheng Z, Chen L, Gao F, Joyce MG, et al. Maturation Pathway from Germline to Broad HIV-1 Neutralizer of a CD4-Mimic Antibody. Cell. 2016;165(2):449-63.

14. Huang J, Kang BH, Ishida E, Zhou T, Griesman T, Sheng Z, et al. Identification of a CD4-Binding-Site Antibody to HIV that Evolved Near-Pan Neutralization Breadth. Immunity. 2016;45(5):1108-21.

15. Gristick HB, von Boehmer L, West AP, Jr., Schamber M, Gazumyan A, Golijanin J, et al. Natively glycosylated HIV-1 Env structure reveals new mode for antibody recognition of the CD4-binding site. Nat Struct Mol Biol. 2016;23(10):906-15.

16. Sajadi MM, Dashti A, Rikhtegaran Tehrani Z, Tolbert WD, Seaman MS, Ouyang X, et al. Identification of Near-Pan-neutralizing Antibodies against HIV-1 by Deconvolution of Plasma Humoral Responses. Cell. 2018;173(7):1783-95.e14.

17. Schommers P, Gruell H, Abernathy ME, Tran MK, Dingens AS, Gristick HB, et al. Restriction of HIV-1 Escape by a Highly Broad and Potent Neutralizing Antibody. Cell. 2020;180(3):471-89.e22.

18. Buchacher A, Predl R, Strutzenberger K, Steinfellner W, Trkola A, Purtscher M, et al. Generation of human monoclonal antibodies against HIV-1 proteins; electrofusion and Epstein-Barr virus transformation for peripheral blood lymphocyte immortalization. AIDS Res Hum Retroviruses. 1994;10(4):359-69.

19. Huber M, Le KM, Doores KJ, Fulton Z, Stanfield RL, Wilson IA, et al. Very few substitutions in a germ line antibody are required to initiate significant domain exchange. J Virol. 2010;84(20):10700-7.

20. Walker LM, Huber M, Doores KJ, Falkowska E, Pejchal R, Julien JP, et al. Broad neutralization coverage of HIV by multiple highly potent antibodies. Nature. 2011;477(7365):466-70.

21. Mouquet H, Scharf L, Euler Z, Liu Y, Eden C, Scheid JF, et al. Complex-type N-glycan recognition by potent broadly neutralizing HIV antibodies. Proc Natl Acad Sci U S A. 2012;109(47):E3268-77.

22. Sok D, Pauthner M, Briney B, Lee JH, Saye-Francisco KL, Hsueh J, et al. A Prominent Site of Antibody Vulnerability on HIV Envelope Incorporates a Motif Associated with CCR5 Binding and Its Camouflaging Glycans. Immunity. 2016;45(1):31-45.

23. MacLeod DT, Choi NM, Briney B, Garces F, Ver LS, Landais E, et al. Early Antibody Lineage Diversification and Independent Limb Maturation Lead to Broad HIV-1 Neutralization Targeting the Env High-Mannose Patch. Immunity. 2016;44(5):1215-26.

24. Longo NS, Sutton MS, Shiakolas AR, Guenaga J, Jarosinski MC, Georgiev IS, et al. Multiple Antibody Lineages in One Donor Target the Glycan-V3 Supersite of the HIV-1 Envelope Glycoprotein and Display a Preference for Quaternary Binding. J Virol. 2016;90(23):10574-86.

25. Alam SM, Aussedat B, Vohra Y, Meyerhoff RR, Cale EM, Walkowicz WE, et al. Mimicry of an HIV broadly neutralizing antibody epitope with a synthetic glycopeptide. Sci Transl Med. 2017;9(381).

26. Freund NT, Wang H, Scharf L, Nogueira L, Horwitz JA, Bar-On Y, et al. Coexistence of potent HIV-1 broadly neutralizing antibodies and antibody-sensitive viruses in a viremic controller. Sci Transl Med. 2017;9(373).

27. Bonsignori M, Kreider EF, Fera D, Meyerhoff RR, Bradley T, Wiehe K, et al. Staged induction of HIV-1 glycan-dependent broadly neutralizing antibodies. Sci Transl Med. 2017;9(381).

28. Jia M, Liberatore RA, Guo Y, Chan KW, Pan R, Lu H, et al. VSV-Displayed HIV-1 Envelope Identifies Broadly Neutralizing Antibodies Class-Switched to IgG and IgA. Cell Host Microbe. 2020;27(6):963-75.e5.

29. Kumar S, Ju B, Shapero B, Lin X, Ren L, Zhang L, et al. A V(H)1-69 antibody lineage from an infected Chinese donor potently neutralizes HIV-1 by targeting the V3 glycan supersite. Sci Adv. 2020;6(38).

30. Walker LM, Phogat SK, Chan-Hui PY, Wagner D, Phung P, Goss JL, et al. Broad and potent neutralizing antibodies from an African donor reveal a new HIV-1 vaccine target. Science. 2009;326(5950):285-9.

31. Bonsignori M, Hwang KK, Chen X, Tsao CY, Morris L, Gray E, et al. Analysis of a clonal lineage of HIV-1 envelope V2/V3 conformational epitope-specific broadly neutralizing antibodies and their inferred unmutated common ancestors. J Virol. 2011;85(19):9998-10009.

32. Sok D, van Gils MJ, Pauthner M, Julien JP, Saye-Francisco KL, Hsueh J, et al. Recombinant HIV envelope trimer selects for quaternary-dependent antibodies targeting the trimer apex. Proc Natl Acad Sci U S A. 2014;111(49):17624-9.

33. Doria-Rose NA, Schramm CA, Gorman J, Moore PL, Bhiman JN, DeKosky BJ, et al. Developmental pathway for potent V1V2-directed HIV-neutralizing antibodies. Nature. 2014;509(7498):55-62.

34. Doria-Rose NA, Bhiman JN, Roark RS, Schramm CA, Gorman J, Chuang GY, et al. New Member of the V1V2-Directed CAP256-VRC26 Lineage That Shows Increased Breadth and Exceptional Potency. J Virol. 2016;90(1):76-91.

35. Cale EM, Gorman J, Radakovich NA, Crooks ET, Osawa K, Tong T, et al. Virus-like Particles Identify an HIV V1V2 Apex-Binding Neutralizing Antibody that Lacks a Protruding Loop. Immunity. 2017;46(5):777-91.e10.

36. Landais E, Murrell B, Briney B, Murrell S, Rantalainen K, Berndsen ZT, et al. HIV Envelope Glycoform Heterogeneity and Localized Diversity Govern the Initiation and Maturation of a V2 Apex Broadly Neutralizing Antibody Lineage. Immunity. 2017;47(5):990-1003.e9.

37. Xiao X, Chen W, Feng Y, Zhu Z, Prabakaran P, Wang Y, et al. Germline-like predecessors of broadly neutralizing antibodies lack measurable binding to HIV-1 envelope glycoproteins: implications for evasion of immune responses and design of vaccine immunogens. Biochem Biophys Res Commun. 2009;390(3):404-9.

38. Zwick MB, Komori HK, Stanfield RL, Church S, Wang M, Parren PW, et al. The long third complementarity-determining region of the heavy chain is important in the activity of the broadly neutralizing anti-human immunodeficiency virus type 1 antibody 2F5. J Virol. 2004;78(6):3155-61.

39. Morris L, Chen X, Alam M, Tomaras G, Zhang R, Marshall DJ, et al. Isolation of a human anti-HIV gp41 membrane proximal region neutralizing antibody by antigen-specific single B cell sorting. PLoS One. 2011;6(9):e23532.

40. Huang J, Ofek G, Laub L, Louder MK, Doria-Rose NA, Longo NS, et al. Broad and potent neutralization of HIV-1 by a gp41-specific human antibody. Nature. 2012;491(7424):406-12.

41. Williams LD, Ofek G, Schätzle S, McDaniel JR, Lu X, Nicely NI, et al. Potent and broad HIV-neutralizing antibodies in memory B cells and plasma. Sci Immunol. 2017;2(7).

42. Pinto D, Fenwick C, Caillat C, Silacci C, Guseva S, Dehez F, et al. Structural Basis for Broad HIV-1 Neutralization by the MPER-Specific Human Broadly Neutralizing Antibody LN01. Cell Host Microbe. 2019;26(5):623-37.e8.

43. Scharf L, Scheid JF, Lee JH, West AP, Jr., Chen C, Gao H, et al. Antibody 8ANC195 reveals a site of broad vulnerability on the HIV-1 envelope spike. Cell Rep. 2014;7(3):785-95.

44. Klein F, Gaebler C, Mouquet H, Sather DN, Lehmann C, Scheid JF, et al. Broad neutralization by a combination of antibodies recognizing the CD4 binding site and a new conformational epitope on the HIV-1 envelope protein. J Exp Med. 2012;209(8):1469-79.

45. Lee JH, Leaman DP, Kim AS, Torrents de la Peña A, Sliepen K, Yasmeen A, et al. Antibodies to a conformational epitope on gp41 neutralize HIV-1 by destabilizing the Env spike. Nat Commun. 2015;6:8167.

46. Falkowska E, Le KM, Ramos A, Doores KJ, Lee JH, Blattner C, et al. Broadly neutralizing HIV antibodies define a glycan-dependent epitope on the prefusion conformation of gp41 on cleaved envelope trimers. Immunity. 2014;40(5):657-68.

47. Huang J, Kang BH, Pancera M, Lee JH, Tong T, Feng Y, et al. Broad and potent HIV-1 neutralization by a human antibody that binds the gp41-gp120 interface. Nature. 2014;515(7525):138-42.

48. van Gils MJ, van den Kerkhof TL, Ozorowski G, Cottrell CA, Sok D, Pauthner M, et al. An HIV-1 antibody from an elite neutralizer implicates the fusion peptide as a site of vulnerability. Nat Microbiol. 2016;2:16199.

49. Kong R, Xu K, Zhou T, Acharya P, Lemmin T, Liu K, et al. Fusion peptide of HIV-1 as a site of vulnerability to neutralizing antibody. Science. 2016;352(6287):828-33.

50. Zhou T, Zheng A, Baxa U, Chuang GY, Georgiev IS, Kong R, et al. A Neutralizing Antibody Recognizing Primarily N-Linked Glycan Targets the Silent Face of the HIV Envelope. Immunity. 2018;48(3):500-13.e6.

51. Schoofs T, Barnes CO, Suh-Toma N, Golijanin J, Schommers P, Gruell H, et al. Broad and Potent Neutralizing Antibodies Recognize the Silent Face of the HIV Envelope. Immunity. 2019;50(6):1513-29.e9.
